# Melatonin and Female Reproduction: An Expanding Universe

**DOI:** 10.3389/fendo.2020.00085

**Published:** 2020-03-06

**Authors:** James M. Olcese

**Affiliations:** Department of Biomedical Sciences, Florida State University College of Medicine, Tallahassee, FL, United States

**Keywords:** melatonin, reproduction, human, fertility, pregnancy, gonads, uterus

## Abstract

For more than a half century the hormone melatonin has been associated with vertebrate reproduction, particularly in the context of seasonal breeding. This association is due in large measure to the fact that melatonin secretion from the pineal gland into the peripheral circulation is a nocturnal event whose duration is reflective of night length, which of course becomes progressively longer during winter months and correspondingly shorter during the summer months. The nocturnal plasma melatonin signal is conserved in essentially all vertebrates and is accessed not just for reproductive rhythms, but for seasonal cycles of metabolic activities, immune functions, and behavioral expression. A vast literature on melatonin and vertebrate biology has accrued over the past 60 years since melatonin's discovery, including the broad topic of animal reproduction, which is far beyond the scope of this human-focused review. Although modern humans in the industrialized world appear in general to have little remaining reproductive seasonality, the relationships between melatonin and human reproduction continue to attract widespread scientific attention. The purpose of this chapter is to draw attention to some newer developments in the field, especially those with relevance to human fertility and reproductive medicine. As the vast majority of studies have focused on the female reproductive system, a discussion of the potential impact of melatonin on human male fertility will be left for others.

## Brief Introduction

By virtue of being a small, amphiphilic, indoleamine molecule, melatonin (5-methoxy-N-acetyl tryptamine) is synthesized *de novo* from serotonin (5-hydroxy-tryptamine), a highly dispersed biologically active molecule in its own right. Historically, melatonin has been considered an endocrine hormone released from the epithalamic pineal gland, which then acts on specific G-protein-coupled melatonin receptors in target tissues of both adults and the fetus (1, 2). More recently, melatonin has been reported to be synthesized in small amounts by a wide variety of animal cells and tissues as well as diverse organisms, including all kingdoms of living organisms [cf. (3, 4)], where it presumably has local paracrine and autocrine actions, some of which are probably independent of specific melatonin receptors (5). Indeed, melatonin has been reported to interact with a great many cellular proteins, including enzymes, channels, transporters, signaling molecules, etc [for a recent comprehensive review, see (6)]. Thus, melatonin is perhaps best defined as both a pineal hormone and a bioactive amine with cellular targets near its site of synthesis in some tissues.

While such generalizations permit the inclusion of many effects, it doesn't remove a number of challenges for the interpretation of the research data involving melatonin. For example, with regard to targets, the reported affinities of the two known human melatonin receptors (in both cell expression systems and *ex vivo*) are in the nanomolar range [cf. (7)], whereas many if not most experimental protocols have employed very pharmacological concentrations to achieve significant effects. Another point to consider is that although plasma melatonin levels are physiologically elevated for many hours at nighttime, protocols often expose tissues or cells to only very short melatonin treatments, which may be physiologically irrelevant. A common response to these concerns is that local concentrations may be quite high and/or constant—especially if there is local constitutive melatonin synthesis. Recent studies suggest that melatonin synthesis by mitochondria may be important for subcellular physiological processes (8). However, there is little experimental evidence that disruption of such local melatonin production has meaningful cellular consequences [in contrast to the removal of plasma melatonin via extirpation of the pineal gland, which has numerous effects on the reproductive axis of laboratory animals - cf. (9)].

The human being is exposed to varying levels of melatonin from conception to death. Much like the hormones thyroxine, insulin or cortisol, the molecule melatonin has a variety of diverse roles to play as a function of developmental life stage (embryo, neonate, adolescent, or adult). It seems likely that many of these actions of melatonin could be permissive or synergistic (like the aforementioned hormones), but there is remarkably scant research into this distinct possibility. A significant step in this direction are the findings of circadian clock gene regulation by melatonin in several tissues of the reproductive axis in both the embryo and adults.

Melatonin has often been described as a chemical output signal of the central circadian oscillator (the hypothalamic suprachiasmatic nuclei, SCN). The clearest support for this statement is the abolition of plasma melatonin rhythmicity following disruption of the neural connectivity between the SCN and the pineal gland. As mentioned earlier, the nocturnal melatonin signal duration is of major importance for physiological seasonality, however, the circadian phasing of the melatonin signal has important ramifications for general circadian functions, including body temperature, endocrine rhythms, and sleep (10). This is in part due to fact that melatonin receptors are expressed in the SCN and can mediate phase-shifting feedback effects of melatonin. Hence, in clinical studies of melatonin actions on the reproductive (or any) system, it is imperative to keep issues such as the timing of melatonin administration (day vs. night) and the duration of the plasma melatonin levels following exogenous melatonin administration in mind. Unfortunately, these considerations are too often overlooked in clinical trials involving melatonin treatments.

Especially in the area of reproductive biology, it is clear that the physiology of animal models is often not comparable to the human state, most especially with regard to ovulatory cycles and the regulation of pregnancy [cf. (11)]. Hence, promising results from melatonin experiments in other species need to be confirmed in clinical trials before one can draw any conclusions of relevance to reproductive medicine. The most obvious historical case in point is the controversy in the late decades of the twentieth century regarding melatonin as an “anti-gonadotrophic” or a “pro-gonadotrophic” hormone (in the human it is physiologically neither, although at high doses there may be some inhibitory effect on ovulation—see more below).

Human reproduction is a challenging object of study for obvious reasons, e.g., population heterogeneity, ethical limits when experimenting with humans, high research costs and appropriate technologies, etc. The quality of clinical data—its statistical power, reproducibility, appropriateness of controls, treatment variations, and so on—make the attainment of firm conclusions thus far about melatonin's normal physiological role in human reproduction difficult. Similarly, validation of proposed pharmacological uses for melatonin or analogs in the treatment of puberty, infertility, menopause, etc has not yet been achieved due to limited published scientific literature. The goal of this chapter is stimulate future research into the relationship of melatonin to human reproductive function in anticipation of generating novel diagnostic and therapeutic tools to improve human health and fertility.

## Melatonin and Puberty

Clinical reports from “pre-melatonin” days (i.e., prior to Aaron Lerner's discovery of the hormone in 1958) identified a potential link between human puberty and pineal tumors. For example, early in the twentieth century Marburg [see his (12) review]—based on reported clinical findings of pineal tumors in children—developed the hypothesis that secretions of the pineal inhibit human reproductive activity. Indeed some clinicians of that generation used pineal extracts to treat precocious puberty (13).

Following the seminal studies of Wurtman et al. (14)—who demonstrated antigonadal effects of melatonin in female rats—the investigation of melatonin's impact on mammalian reproduction rapidly expanded [cf. (15)]. However, with regard to human puberty and its regulation by melatonin, conflicting reports appeared in the later quarter of the twentieth century. Whereas, some groups found higher plasma melatonin levels associating with prepubertal and delayed pubertal conditions (16, 17) and inversely lower levels of melatonin after puberty or in cases of precocious puberty (18–20), numerous other groups found no significant differences between normal and disordered puberty (21–24). These discrepancies have led to skepticism among twenty-first century clinicians regarding the importance of melatonin in normal pubertal development. In both young males and females, the puberty-related decline of high childhood melatonin levels has been correlated more to advancing Tanner stages than to chronological age (25), but no clear causative basis for this relationship has been established for humans.

In view of the circadian secretion of melatonin and the circadian nature of pituitary hormone levels during puberty and in adults, it has long been suggested that melatonin regulates human reproductive cycles. The pulsatile release of GnRH and hence gonadotropin pulse frequency is highest during the night during puberty (26) and the monthly surge of LH and FSH secretion at ovulation also occurs mainly during the latter hours of the dark phase (27, 28). To what extent the temporal coincidence of hypothalamic secretions with melatonin release simply reflects coordinated downstream activation of neural pathways under control of the central circadian oscillator in the SCN as opposed to explicit regulation of the neuroendocrine axis by melatonin remains unclear—most likely both pathways play some role.

## Melatonin and the Female Reproductive Cycle

Melatonin receptors have been demonstrated in a variety of cell types in the female reproductive tract uterus. As shown in Table 1 the majority of studies demonstrate dual expression of both MT1 and MT2 receptors. Hence, one should consider all of these cells to be potential targets for melatonin action.

**Table 1:** Melatonin binding sites in the human female reproductive system.

**Organ**	**Cells**	**Receptor type**	**Response**	**References**
Ovary	Granulosa/luteal	nd	↑ P4	(29–31)
		MT1, MT2	↓ P4	(32, 33)
		nd	↓ BMP6 signaling	(34)
		nd	↓ oxid. Stress	(31)
Uterus	Myometrium	MT1, MT2	↑ contractility	(35, 36)
				(37)
Breast	Glandular epithelium	MT1	↓ERα transcriptional activity	cf. (38)
Placenta	Choriocarcinoma	MT1, MT2	↓hCG secretion	(39)
	Trophoblast	MT1, MT2	↓Inflammation, autophagy	(40, 41) (42, 43)

Some 30 years ago, Brzezinski et al. demonstrated that human preovulatory follicular fluid contained melatonin at levels higher than plasma melatonin levels (44). This was subsequently confirmed and later shown to vary inversely with day length and concomitantly with follicular progesterone (P4) levels (45, 46), suggesting preferential uptake of circulating melatonin by the ovary. Nakamura et al. (47) subsequently found that larger preovulatory follicles had higher melatonin levels than smaller immature follicles. Later observations that increasing oral doses of melatonin results in significantly elevated melatonin concentrations in follicular fluid of women volunteers (48) also support this view. Several reports followed in which melatonin was shown to modulate progesterone production by cultured human granulosa/luteal cells (29, 49, 50). More recently, the effects of melatonin on cultured human granulosa/luteal cells has been extended to include synergism with hCG—albeit at very high melatonin concentrations (51).

Interestingly, when combined with progesterone, melatonin at high doses is able to suppress human ovulation (52). As will be discussed later, rising progesterone levels during human pregnancy (when ovulation is strongly suppressed) are accompanied by rising plasma melatonin levels. It could be insightful to assess the effects of progesterone on melatonin receptor expression in the human ovary and other reproductive tissues.

Among 61 women undergoing assisted reproductive therapy (ART) treatment cycles it was reported that a positive correlation exists between follicular melatonin levels and markers of ovarian reserve, e.g., anti-Muellerian hormone and baseline FSH levels (53). These authors also found a similar correlation between follicular fluid melatonin levels and *in vitro* fertilization (IVF) outcomes and oocyte quality. Similarly, Zheng et al. (54) found a significant positive correlation between follicular fluid melatonin concentrations and antral follicle count in women undergoing *in vitro* fertilization—also consistent with a supportive or protective action of melatonin on ovarian cycle progression.

These results have motivated a number of studies into the potential benefit of pharmacological melatonin supplementation in the treatment of infertility [cf. (55) for review]. Although the etiology of infertility is complex and not fully clarified, a recurring aspect appears to be excessive production of reactive oxygen species in the follicular fluid (56). In an oft-cited study by Tamura et al. (57), it was reported that when patients were given 3 mg of melatonin orally in the evening from the fifth day of the previous menstrual cycle until the day of oocyte retrieval, intra-follicular concentrations of melatonin rose 4-fold. Markers of intra-follicular oxidative damage were decreased after melatonin treatment compared to those in the prior cycle, suggesting that melatonin treatment reduces intra-follicular oxidative stress. These investigators then assessed the clinical outcomes of 115 patients who failed to become pregnant in the previous IVF-ET cycle with a low fertilization rate (< 50%). In the 56 patients treated with melatonin, the fertilization rate (50.0 ± 38.0%) was markedly improved compared with the previous IVF-ET cycle (20.2 ± 19.0%), and 11 of 56 patients (19.6%) achieved pregnancy. In contrast, in the 59 control patients, the fertilization rate (22.8 ± 19.0 vs. 20.9 ± 16.5%) was not significantly changed, and only 6 of 59 patients (10.2%) achieved pregnancy. These intriguing findings are consistent with the view that pharmacological melatonin administration increases intra-follicular melatonin concentrations, reduces intra-follicular oxidative damage and may have a beneficial effect on fertilization and pregnancy rates during ART. In a recent report, similar benefits were found in 40 women with idiopathic infertility who were administered pharmacological melatonin and who subsequently showed improved intrafollicular oxidative capacity and oocyte quality in IVF protocols (58).

These data point to a potential benefit of melatonin in the process of oogenesis. In this regard, the high levels of melatonin required for effects seem consistent with high follicular fluid concentrations of melatonin. Some investigators have also proposed that follicular granulosa cells have the capability for local melatonin synthesis (59, 60), which if confirmed would add new insight into melatonin's role as a paracrine modulator in the reproductive system of humans.

Many female reproductive hormones undergo 24-h rhythms under both standard sleep-wake cycles and under constant routine conditions, indicating that they are under endogenous circadian control (61, 62). Interestingly, these rhythms are robust in the early follicular phase but not in the luteal phase of the menstrual cycle, which is largely under the control of high luteal progesterone secretions. Perturbations of the human circadian system (e.g., from shift work) are known to disrupt reproductive cycles [cf. (63) for review]. However, data are lacking on the potential role of melatonin to the etiology of these disruptions, despite initial early findings in one small study (64) that demonstrated a high incidence of irregular menstrual cycles in night workers whose melatonin levels were significantly suppressed.

## Melatonin and Pregnancy

Following earlier research on maternal transfer of melatonin to the fetus and neonatal development of pineal melatonin rhythmicity in the 1980s and 1990s (65–67), a period of relative quiescence welcomed the transition to a new millennium. However, a growing number of studies in the past decade have focused on the action of melatonin on the placenta and fetal development as well as circadian roles for melatonin before and after parturition.

It is now relatively well-established that a prime entrainment signal from the maternal circulation to fetus is melatonin (68), which crosses the placenta (69), and can bind to melatonin receptors in numerous fetal tissues (70).

Melatonin receptor (MT1 and MT2) transcripts and proteins have also been detected in human placentae (39–41) and subsequently shown to be expressed throughout pregnancy, albeit with declining levels after the first trimester (71). In addition, mRNA and protein expression of the melatonin-synthesizing enzymes, AANAT and HIOMT, have also been reported (40, 41), leading to the view that in addition to the rising plasma melatonin levels during pregnancy (72), local production of melatonin may serve an additional paracrine role. One target may be the placental trophoblast cells, which secrete the “pregnancy hormone” human chorionic gonadotropin (hCG). In two separate laboratories, it was found that high micromolar to millimolar concentrations of melatonin *in vitro* significantly elevated hCG release by human trophoblast cells (40, 71). More recently, the latter group also reported that melatonin at these high levels protects trophoblast cells *in vitro* against hypoxia/reoxygenation–induced inflammation and autophagy (42).

Another potential target for melatonin may be the vascularization of the placenta in early pregnancy through remodeling of the maternal uterine spiral arteries, a process that appears to be defective in preeclampsia—a leading cause of maternal mortality, especially in developing countries. Placental and systemic oxidative stress is considered to be a major underlying mechanism of pathology in preeclampsia (73). With a view toward melatonin's antioxidant properties, it is striking that blood levels and placental synthesis of melatonin decline significantly in women with severe preeclampsia (74–76). In a meta-analysis by Dou et al. (77), these data were corroborated and melatonin levels were found to correlate with the severity of the disease. In terms of a possible beneficial effect of melatonin on placental tissues, Hannan et al. (78) demonstrated that melatonin *in vitro* upregulated antioxidant response genes in human placental trophoblasts as well as in umbilical vein endothelial cells, albeit only at extremely high (1 mM) levels. A recent pilot clinical study by Hobson et al. (79) essentially corroborated these *in vitro* findings. These authors reported modest improvements in the duration of pregnancy in a small cohort of women with preeclampsia who had taken 10 mg oral melatonin three times per day from recruitment until delivery. While it seems highly unlikely that these pharmacological concentrations of melatonin reflect *in vivo* circumstances, these results non-etheless open the door for new therapeutic possibilities to improve clinical outcomes for women with preeclampsia.

## Melatonin and Parturition

Given its proven role as an endocrine signal of night time duration (7, 80) it was not unexpected to find an influence of melatonin on the timing of parturition. Takayama et al. (81) showed that female rats whose endogenous melatonin was eliminated by pinealectomy had no disturbances in estrous cyclicity or in their ability to become pregnant, but they failed to deliver their young exclusively during the daytime (early daytime is the normal birthing phase for nocturnal animals, such as rodents). Instead, the rats gave birth randomly across the 24-h light-dark cycle. However, evening administration of melatonin (i.e., at the time when endogenous levels would normally increase) was effective in restoring the normal daytime birth pattern. Importantly, melatonin was ineffectual when given in the morning or continuously. This strongly points to the ***timing*** of birth in the rat being under circadian control, and that melatonin may serve as a key circadian “gating” signal for this event. These data suggest that the clock may play a subtle, but important role in the reproductive process; however, care must be taken when extrapolating rat data to the human as we are largely diurnal (day-time active), whereas the majority of laboratory rodents are nocturnal (night-time active).

The precise mode of action of melatonin in the mammalian uterus, while still not completely understood, is clearly species-specific. Some earlier reports with rodents (82, 83) showed direct *inhibitory* effects of pharmacological doses of melatonin on uterine contractility as well as the presence of melatonin-specific binding sites in the uterus (84). There are further reports of the inhibitory effects of melatonin on prostaglandin synthesis in various rodent tissues (85, 86). Melatonin has also been shown to modulate calcium signaling in various tissues, including vascular smooth muscle, often via synergistic actions with other receptor-mediated processes (7). Again, care must also be taken when extrapolating data gathered from nocturnal species, such as the laboratory mouse (C57/Bl6), as these and other strains do not produce endogenous melatonin and their parturition physiology is vastly different from that of the human female.

In contrast to the nocturnal rodent, human labor, and delivery are statistically more common during the night phase (87, 88). In view of its nocturnal secretion pattern and the reported effects of melatonin on uterine contractions in other mammals, it seemed reasonable to explore whether melatonin may act as the “temporal gate” in contributing to the contractions that underlie human parturition.

**Figure 1:**
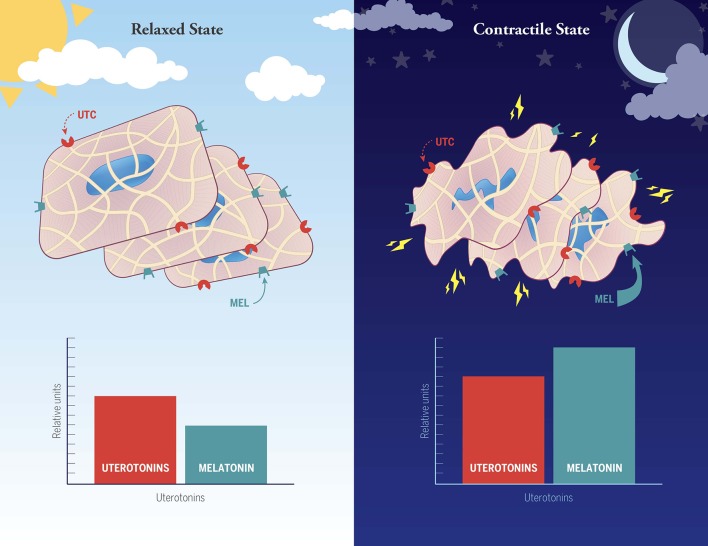
Hypothesized involvement of melatonin with other non-specified uterotonins (e.g., oxytocin, prostaglandins, cytokines) in the establishment of the well-known day night difference in human myometrial contractions during normal late term pregnancy and labor. In this working model the uterine smooth muscle cells are suggested to be in a relaxed state during the daytime because of low levels of melatonin and other unspecified uterotonins (note: the histograms are not meant to be quantitative, but rather to depict relative differences between day and night). High nocturnal uterine contractility is proposed to arise as a synergistic response between uterotonic factors and melatonin, the latter of which is markedly elevated at night. Suppression of nocturnal melatonin levels in late term pregnant women has been demonstrated to significantly reduce uterine contractility (89).

Data from our laboratory (35, 37) have uncovered a significant positive synergistic action of melatonin and oxytocin (OT) on human myometrial smooth muscle cell contractions *in vitro* in which melatonin results in striking amplification of OT-induced IP_3_ signaling and OT-induced contractions. These findings may explain the high level of nocturnal uterine contractions found in late term human pregnancy that lead to nocturnal labor (Figure 1). More recently, we have also identified a synergistic action of melatonin and OT on myometrial smooth muscle cell induction of the core circadian gene *hBMAL1* (90). BMAL1 is a transcription factor at the core of the circadian system (91, 92) as it serves to regulate the expression of genes whose promoters contain the E-box motif, which includes the melatonin receptors. OT analogs are important tools in obstetrical practice. Continuous infusion of OT agonists is commonly used to induce labor, while OT antagonists are now used to prolong pregnancy in cases of preterm labor. However, prolonged labor induction by application of continuous OT is only effective when high amounts of the hormone are given [due to receptor “desensitization”- (93)]. Unfortunately, continuous OT administration is often accompanied by serious side effects, including fetal distress, uterine rupture, postpartum atony and bleeding. Discovery of a synergism between OT and melatonin signaling (35, 37) could eventually lead to the development of new melatonin + low dose OT medicinal combinations for labor induction without the considerable side-effects of high OT administration. Conversely, studies employing the well-known inhibitory effect of light of circulating melatonin levels have provided corroborating evidence that the nocturnal uterine contractions common to late pregnancy are under melatonin control (94, 95).

A strong, parallel upregulation of the melatonin MTNR1B receptor and oxytocin receptor (OTR) protein in the myometrium of ***laboring*** pregnant, as compared to non-laboring pregnant women has also been demonstrated (35). Parallel trends were noted for *MTNR1A* and *MTNR1B* mRNA expression and for melatonin-binding to these same samples (89) implying melatonin receptor suppression throughout most of gestation with activation (de-suppression) at the end of pregnancy in preparation for parturition. Although uterine quiescence is thought to be a key function of progesterone during pregnancy [e.g., (96, 97)], it is unclear whether melatonin receptor activation in the human myometrium at term pregnancy involves changes in progesterone signaling. Interestingly, in preliminary studies of biopsies from women who entered preterm labor, melatonin receptor protein expression was detected in all samples (98), leading to the fascinating possibility that premature expression of myometrial melatonin receptors may predispose a woman to contractions and preterm labor (99).

## Melatonin in Reproductive Aging

In contrast to early childhood, when high melatonin levels are correlated with low gonadotropin secretion, the presence of low melatonin levels in elderly people appears to be correlated with reproductive aging, i.e., high gonadotropin secretion (100, 101). It is well-established that plasma melatonin levels in elderly individuals are reduced and that the circadian timing of the nocturnal melatonin peak is advanced (102–105).

The normal cessation of female reproductive fertility (menopause) is determined by the inability of the ovaries to produce viable follicles and changes in hormonal secretion that leads to failure of menstrual cycles. Thus, clinically, ovarian aging is characterized by a diminished follicular reserve, which correlates with elevated gonadotropin secretion from the anterior pituitary.

An earlier report of mitigation of depression, and improved mood and sleep quality following melatonin administration to perimenopausal and postmenopausal women (106) could not be confirmed in a study by Amstrup et al. (107) which found no significant effect on quality of life or sleep quality in 81 postmenopausal women who were given pharmacological melatonin nightly for 1 year. However, these authors did report a non-significant trend toward improved sleep quality in a subgroup of melatonin-treated women who had sleep disturbances at initial baseline. Toffol et al. (108) showed that postmenopausal women have lower nighttime serum melatonin levels than perimenopausal women; however, they found no correlations between serum melatonin and FSH or estradiol levels, Beck Depression Inventory score, State-Trait Anxiety Inventory score, BNSQ insomnia score, BNSQ sleepiness score, subjective sleep score, climacteric vasomotor score, or quality of life. The apparent discrepancies in the aforementioned studies is probably reconcilable, since in the Bellipanni and Amstrup investigations pharmacological levels of melatonin (3 mg/night for 6–12 months) were administered, while the Toffol study analyzed physiological and psychological correlations with naturally reduced endogenous melatonin levels. More recently however, long term pharmacological melatonin administration was shown to reduce psychosomatic symptoms in postmenopausal women after 12 months of treatment in a double-blind, placebo study (109). This is consistent with numerous previous studies on the use of pharmacological melatonin in the treatment of sleep disturbances in elderly men and women [cf. (110)].

Some studies have proposed a role for melatonin in ovarian aging, given the supportive and pleiotropic effects of melatonin on ovarian activities, including suppression of oxidative stress, protection of mitochondrial integrity, etc (48, 111, 112). However, as most of the research to date has been in rodents [cf. (113, 114)] a clear etiological relationship between declining endogenous melatonin levels and human menopause has not been adequately demonstrated, nor have sufficiently powered clinical trials with melatonin administration to perimenopausal women been reported.

## Summary and Future Perspectives

To summarize this short overview of melatonin's association with human female reproduction and fertility, melatonin does not have a strong impact on human puberty, although in this regard its contributions as an endocrine output of the circadian clock need further careful study. Its impact on oogenesis and ovulation, while modest, could still be valuable in the development of new treatments for certain forms of female infertility. Along similar lines, pharmacological melatonin, or analogs may ultimately find application in placental therapeutics, e.g., for treating placental inflammation, oxidative stress, and preeclampsia. And finally, melatonin receptors in the human uterine smooth muscles may offer a surprising new target for the management of labor, both term labor and preterm labor.

There is thus far little substantiated information on the potential association between clinical syndromes involving the reproductive system and melatonin deficits (or excess), or melatonin receptor polymorphisms. Luboshitzky et al. (115) documented increased excretion of the major melatonin metabolite (6-sulfatoxymelatonin) in women with PCOS, although whether this was a consequence of increased or decreased plasma melatonin levels was not apparent. More recently, in a pilot study Tagliaferri et al. (116) administered pharmacological doses of oral melatonin for 6 months to 40 women with PCOS and reported significant improvements in menstrual cyclicity and normalization of androgen balance. A similar reduction of PCOS-related hirsutism and androgen levels after 12 weeks of melatonin supplementation was also found in a recent investigation by Jamilian et al. (117). Thus, the therapeutic use of melatonin in women with PCOS-related conditions shows promise, and should be explored further. Interestingly, Song et al. (118) identified significant gene polymorphism differences in a region of the melatonin type 1 receptor (MTNR1a) between women with polycystic ovarian syndrome (PCOS), but no associated phenotypic differences were seen. Whether other polymorphisms in the human melatonin receptor can be related to other reproductive disorders remains a fascinating though largely uncharted territory.

While research into the potential roles of melatonin in human reproductive physiology continues to expand our intellectual universe after six decades, some common features are apparent. Firstly, melatonin can potentially reach every cell of the body, conveying both circadian information (via plasma melatonin rhythms) and serving as a paracrine modulator of local oxidative state, inflammatory responses, autophagy, etc (e.g., in the ovary and placenta). Some of these actions are likely to be melatonin receptor-dependent, while others may be receptor-independent. In some cases, melatonin may serve as a permissive or synergistic signal, affecting the response of tissues to other molecules (e.g., oxytocin in the uterus). As an ancient molecule that has taken membership in a wide array of cellular processes in all kingdoms of living organisms over the eons of terrestrial biological evolution, melatonin's involvement in human reproduction is best described to be subtle, diverse, and essential. For example, from *in utero* fetal programming to the timing of parturition, and from influences on metabolism in key reproductive tissues to modulation of neuroendocrine rhythms, melatonin appears to make contributions to all of these processes.

On a closing note, it is critically important to again make the distinction between physiological effects of melatonin and pharmacological consequences of melatonin administration. This warning is of course not unique for melatonin—consider for example another circadian hormone like cortisol. Similarly, when evaluating target effects of melatonin one must remain attentive to species-specific differences in the responsiveness of any tissue to rhythmic and also non-rhythmic levels of melatonin. Developmental stage, gender differences and genetic variabilities all can affect how a reproductive tissue will respond to melatonin. In this regard, it behooves the melatonin researchers of the future to maintain an open mind and an eye for the unexpected. Clearly, melatonin and/or novel analogs of melatonin will eventually find their place in the armamentarium of reproductive medicine in ways that we probably can't even imagine.

## Author Contributions

The author confirms being the sole contributor of this work and has approved it for publication.

### Conflict of Interest

The author declares that the research was conducted in the absence of any commercial or financial relationships that could be construed as a potential conflict of interest.

## References

[1] Thomas L Drew JE Abramovich DR Williams LM. The role of melatonin in the human fetus. Intl J Molec Med. (1998) 1:539–43. 10.3892/ijmm.1.3.539 9852259

[2] Cecon E Oishi A Jockers R Melatonin receptors: molecular pharmacology and signaling in the context of system bias. Brit J Pharmacol. (2018) 175:3263–80. 10.1111/bph.13950 28707298 PMC6057902

[3] Reiter RJ Rosales-Corral SA Manchester LC Tan DX. Peripheral reproductive organ health and melatonin: ready for prime time. Int J Mol Sci. (2013) 14:7231–72. 10.3390/ijms14047231 23549263 PMC3645684

[4] Acuna-Castroviejo D Escames G Venegas C Díaz-Casado ME Lima-Cabello E López LC. Extrapineal melatonin: sources, regulation, and potential functions. Cell Molec Life Sci. (2014) 71:2997–3025. 10.1007/s00018-014-1579-2 24554058 PMC11113552

[5] Manchester LC Pilar Terron M Flores LJ Koppisepi S. Medical implications of melatonin: receptormediated and receptor-independent actions. Adv Med Sci. (2007) 52:11–28. 18217386

[6] Liu L Labani N Cecon E Jockers R. Melatonin target proteins: too many or not enough? Front Endocrinol. (2019) 10:791. 10.3389/fendo.2019.00791 31803142 PMC6872631

[7] Dubocovich ML Delagrange P Krause DN Sugden D Cardinali DP Olcese J International union of basic and clinical pharmacology. LXXNomenclature V. classification, and pharmacology of G protein-coupled melatonin receptors. Pharmacol Rev. (2010) 62:343–80. 10.1124/pr.110.002832 20605968 PMC2964901

[8] Suofu Y Li W Jean-Alphonse FG Jia J Khattar NK Li J. Dual role of mitochondria in producing melatonin and driving GPCR signaling to block cytochrome c release. Proc Natl Acad Sci USA. (2017) 114:E7997–8006. 10.1073/pnas.1705768114 28874589 PMC5617277

[9] Arendt J. (1995). Melatonin and the Mammalian Pineal Gland. London: Chapman & Hall.

[10] López-Canul M Min SH Posa L De Gregorio D Bedini A Spadoni G. Melatonin MT1 and MT2 receptors exhibit distinct effects in the modulation of body temperature across the light/dark cycle. Int J Mol Sci. (2019) 20:2452. 10.3390/ijms20102452 31108968 PMC6566544

[11] Mitchell BF Taggart MJ. Are animal models relevant to key aspects of human parturition? Amer J Physiol Regul Integr Comp Physiol. (2009) 297:R525–45. 10.1152/ajpregu.00153.2009 19515978

[12] Marburg O Die Physiologie der Zirbeldruese (*Glandula pinealis*). Handbuch der Normalen Patholog Physiol. (1930) 13:493–590.

[13] Engel P Die physiologische und pathologische Bedeutung der Zirbeldruese. Ergebn Inn Med. (1936) 50:116–71. 10.1007/978-3-642-90691-6_3

[14] Wurtman RJ Axelrod J Chu EW. Melatonin, a pineal substance: its effect on the rat ovary. Science. (1963) 141:277–80. 10.1126/science.141.3577.277 14002083

[15] Johnston JD Skene DJ. Regulation of mammalian neuroendocrine physiology and rhythms by melatonin. J Endo. (2015) 226:187–98. 10.1530/JOE-15-0119 26101375

[16] Silman RE Leone RM Hooper RJ Preece MA. Melatonin, the pineal gland and human puberty. Nature. (1979) 282:301–3. 10.1038/282301a0 503201

[17] Waldhauser F Weiszenbacher G Frisch H Zeitlhuber U Waldhauser M Wurtman RJ. Fall in nocturnal serum melatonin during prepuberty and pubescence. Lancet. (1984) 1:362–5. 10.1016/S0140-6736(84)90412-4 6141425

[18] Attanasio A Borrelli P Gupta D. Circadian rhythms in serum melatonin from infancy to adolescence. J Clin Endocrinol Metabol. (1985) 61:388–90. 10.1210/jcem-61-2-388 4008613

[19] Waldhauser F Boepple PA Schemper M Mansfield MJ Crowley WF. Serum melatonin in central precocious puberty is lower than in age-matched prepubertal children. J Clin Endocrinol Metab. (1991) 73:793–6. 10.1210/jcem-73-4-793 1909703

[20] Puig-Domingo M Webb SM Serrano J Peinado MA Corcoy R Ruscalleda J. Brief report: melatonin-related hypogonadotropic hypogonadism. N Engl J Med. (1992) 327:1356–9. 10.1056/NEJM199211053271905 1406837

[21] Kennaway DJ Mcculloch G Matthews CD Seamark RF. Plasma melatonin, luteinizing hormone, follicle-stimulating hormone, prolactin, and corticoids in two patients with pinealoma. J Clin Endocrinol Metab. (1979) 49:144–5 10.1210/jcem-49-1-144 447812

[22] Arendt J Melatonin assays in body fluids. J Neural Trans Suppl. (1978) 13:265–78. 288853

[23] Tamarkin L Abastillas P Chen HC McNemar A Sidbury JB. The daily profile of plasma melatonin in obese and Prader-Willi syndrome children. J Clin Endocrinol Metab. (1982) 55:491–495. 7096537 10.1210/jcem-55-3-491

[24] Cavallo A. Melatonin and human puberty: current perspectives. J Pineal Res. (1993) 15:115–21. 10.1111/j.1600-079x.1993.tb00517.x 8106956

[25] Salti R Galluzzi F Bindi G Perfetto F Tarquini R Halberg F. Nocturnal melatonin patterns in children. J Clin Endocrinol Metab. (2000) 85:2137–44. 10.1210/jcem.85.6.6656 10852442

[26] Grumbach MM. The neuroendocrinology of human puberty revisited. Hormone Res. (2002) 57:2–14. 10.1159/000058094 12065920

[27] Cahill DJ Wardle PG Harlow CR Hull MG. Onset of the preovulatory luteinizing hormone surge: diurnal timing and critical follicular prerequisites. Fertil Steril. (1998) 70:56–9. 10.1016/S0015-0282(98)00113-7 9660421

[28] Russo KA La JL Stephens SB Poling MC Padgaonkar NA. Circadian control of the female reproductive axis through gated responsiveness of the RFRP-3 system to VIP signaling. Endocrinology. (2015) 156:2608–18. 10.1210/en.2014-1762 25872006 PMC4475714

[29] Webley GE Luck MR. Melatonin directly stimulates the secretion of progesterone by human and bovine granulosa cells *in vitro*. J Reprod Fertil. (1986) 78:711–7. 10.1530/jrf.0.0780711 3806524

[30] Webley GE Luck MR Hearn JP. Stimulation of progesterone secretion by cultured human granulosa cells with melatonin and catecholamines. J Reprod Fertil. (1988) 84:669–77. 10.1530/jrf.0.0840669 3199386

[31] Taketani T Tamura H Takasaki A Lee L Kizuka F Tamura I Protective effects of melatonin in progesterone production by human luteal cells. J Pineal Res. (2011) 51:207–13. 10.1111/j.1600-079X.2011.00878.x 21585519

[32] Niles LP Wang J Shen L Lobb DK Younglai EV. Melatonin receptor mRNA expression in human granulosa cells. Mol Cell Endocrinol. (1999) 156:107–10. 10.1016/S0303-7207(99)00135-5 10612428

[33] Woo MM Tai CJ Kang SK Nathwani PS Pan SF Leung PC. Direct action of melatonin in human granulosa-luteal cells. J Clin Endocrinol Metab. (2001) 86:4789–97. 10.1210/jcem.86.10.7912 11600542

[34] Otsuka F. Interaction of melatonin and BMP-6 in ovarian steroidogenesis. Vitamins Hormones. (2018) 107:137–53. 10.1016/bs.vh.2018.01.012 29544628

[35] Sharkey J Puttaramu R Word RA Olcese J. Melatonin synergizes with oxytocin to enhance contractility of human myometrial smooth muscle cells. J Clin Endocrinol Metab. (2009) 94:421–7. 10.1210/jc.2008-1723 19001515 PMC2730229

[36] Schlabritz-Loutsevitch N Hellner N Middendorf R Müller D Olcese J. The human myometrium as a target for melatonin. J Clin Endocrinol Metab. (2003) 88:908–13. 10.1210/jc.2002-020449 12574232

[37] Sharkey J Cable C Olcese J Melatonin sensitizes human myometrial cells to oxytocin in a PKCα/ERK-dependent manner. J Clin Endocrinol Metab. (2010) 95:2902–8. 10.1210/jc.2009-2137 20382690 PMC2902072

[38] Hill SM Belancio VP Dauchy RT Xiang S Brimer S Mao L. Melatonin: an inhibitor of breast cancer. Endocrine-Related Cancer. (2015) 22:R183–204. 10.1530/ERC-15-0030 25876649 PMC4457700

[39] Lanoix D Ouellette R Vaillancourt C. Expression of melatoninergic receptors in human placental choriocarcinoma cell lines. Human Reprod. (2006) 21:1981–9. 10.1093/humrep/del120 16632463

[40] Iwasaki S Nakazawa K Sakai J Kometani K Iwashita M Yoshimura Y. Melatonin as a local regulator of human placental function. J Pineal Res. (2005) 39:261–5. 10.1111/j.1600-079X.2005.00244.x 16150106

[41] Lanoix D Beghdadi H Lafond J Vaillancourt C. Human placental trophoblasts synthesize melatonin and express its receptors. J Pineal Res. (2008) 45:50–60. 10.1111/j.1600-079X.2008.00555.x 18312298

[42] Sagrillo-Fagundes L Salustiano EMA Ruano R Markus RP Vaillancourt C. Melatonin modulates autophagy and inflammation protecting human placental trophoblast from hypoxia/reoxygenation. J Pineal Res. (2018) 65:e12520. 10.1111/jpi.12520 30091210

[43] Sagrillo-Fagundes L Bienvenue-Pariseault J Vaillancourt C. Melatonin: the smart molecule that differentially modulates autophagy in tumor and normal placental cells. PLoS ONE. (2019) 14:e0202458 10.1371/journal.pone.0202458 30629581 PMC6328125

[44] Brzezinski A Seibel MM Lynch HJ Deng MH Wurtman RJ. Melatonin in human preovulatory follicular fluid. J Clin Endocrinol Metab. (1987) 64:865–7. 10.1210/jcem-64-4-865 3818907

[45] Roennberg L Kauppila A Leppaeluoto J Martikainen H Vakkuri O Circadian and seasonal variation in human preovulatory follicular fluid melatonin concentration. J Clin Endocrinol Metab. (1990) 71:492–6. 10.1210/jcem-71-2-493 2380343

[46] Yie SM Brown GM Liu GY Collins JA Daya S Hughes EG. Melatonin and steroids in human pre-ovulatory follicular fluid: seasonal variations and granulosa cell steroid production. Hum Reprod. (1995) 10:50–5. 10.1093/humrep/10.1.50 7745070

[47] Nakamura Y Tanura H Takayama H Kato H Increased endogenous level of melatonin in preovulatory human follicles does not directly influence progesterone production. Fertil Steril. (2003) 80:1012–6. 10.1016/S0015-0282(03)01008-2 14556825

[48] Tamura H Takasaki A Taketani T Tanabe M Lee L Tamura I. Melatonin and female reproduction. J Obstet Gynaecol Res. (2014) 40:1–11. 10.1111/jog.12177 24118696

[49] Brzezinski A Schenker JG Fibich T Laufer N Cohen M. Effects of melatonin on progesterone production by human granulosa lutein cells in culture. Fertil Steril. (1992) 58:526–9. 10.1016/S0015-0282(16)55257-1 1521647

[50] Schaeffer HJ Sirotkin AV Melatonin and serotonin regulate the release of insulin-like growth factor-1, oxytocin and progesterone by cultured human granulosa cells. Exp Clin Endocrinol Diabetes. (1997) 105:109–12. 10.1055/s-0029-1211736 9137942

[51] Scarinci E Tropea A Notaristefano G Arena V Alesiani O Fabozzi SM. Hormone of darkness and human reproductive process: direct regulatory role of melatonin in human corpus luteum. J Endocrinol Invest. (2019) 42:1191–97. 10.1007/s40618-019-01036-3 30912058

[52] Voordouw B Euser R Verdonk R Alberda B deJong F Drogendijk A. Melatonin and melatonin-progestin combinations alter pituitary-ovarian function in women and can inhibit ovulation. J Clin Endocrinol Metab. (1992) 74:108–17. 10.1210/jcem.74.1.1727807 1727807

[53] Tong J Sheng S Sun Y Li H Li WP Zhang C. Melatonin levels in follicular fluid as markers for IVF outcomes and predicting ovarian reserve. Reproduction. (2017) 153:443–51. 10.1530/REP-16-0641 28062641

[54] Zheng M Tong J Wei-Ping L Chen ZJ Zhang C. Melatonin concentration in follicular fluid is correlated with antral follicle count (AFC) and *in vitro* fertilization outcomes in women undergoing assisted reproductive technology (ART) procedures. Gynecol Endocrinol. (2018) 34:446–50. 10.1080/09513590.2017.1409713 29185361

[55] Fernando S Rombauts L. Melatonin: shedding light of infertility? A review of recent literature. J Ovar Res. (2014) 7:98 10.1186/s13048-014-0098-y 25330986 PMC4209073

[56] Lee KS Joo BS Na YJ Yoon MS Choi OH Kim WW. Relationships between concentrations of tumor necrosis factor-alpha and nitric oxide in follicular fluid and oocyte quality. J Assist Reprod Genet. (2000) 17:222–228. 10.1023/A:1009495913119 10955247 PMC3455467

[57] Tamura H Takasaki A Taketani T Tanabe M Kizuka F Lee L. The role of melatonin as an antioxidant in the follicle. J Ovarian Res. (2012) 5:5. 10.1186/1757-2215-5-5 22277103 PMC3296634

[58] Espino J Macedo M Lozano G Ortiz A Rodriguez C Rodriguez AB. Impact of melatonin supplementation in women with unexplained infertility undergoing fertility treatment. Antioxidants. (2019) 8:338. 10.3390/antiox8090338 31450726 PMC6769719

[59] Tamura H Nakamura Y Korkmaz A Manchester LC Tan DX Sugino N Melatonin and the ovary: physiological and pathological implications. Fertil Steril. (2009) 92:328–343. 10.1016/j.fertnstert.2008.05.016 18804205

[60] Reiter RJ Tan DX Fuentes-Broto L. Melatonin: a multitasking molecule. Prog Brain Res. (2010) 181:127–51. 10.1016/S0079-6123(08)81008-4 20478436

[61] Simonneaux V Bahougne T. Daily rhythms count for female fertility. Best Pract Res Clin Endocrinol Metab. (2017) 31: 505–19. 10.1016/j.beem.2017.10.012 29223284

[62] Rahman SA Grant LK Gooley JJ Rajaratnam SMW Czeisler CA. Endogenous circadian regulation of female reproductive hormones. J Clin Endocrinol Metab. (2019) 104:6049–59. 10.1210/jc.2019-00803 31415086 PMC6821202

[63] Gamble KL Resuehr D Johnson CH. Shift work and circadian dysregulation of reproduction. Front Endocrinol. (2013) 4:92. 10.3389/fendo.2013.00092 23966978 PMC3736045

[64] Miyauchi F Nanjo K Otsuka K. Effects of night shift on plasma concentrations of melatonin, LH. FSH and prolactin, and menstrual irregularity. Sangyo Igaku. (1992) 34:545–50 10.1539/joh1959.34.545 1460786

[65] Davis FC Mannion J. Entrainment of hamster pup circadian rhythms by prenatal melatonin injections to the mother. Amer J Physiol. (1988) 255:R439–48. 10.1152/ajpregu.1988.255.3.R439 3414839

[66] Davis FC. Melatonin: role in development. J Biol Rhythms. (1997) 12:498–508. 10.1177/074873049701200603 9406023

[67] Kennaway DJ Stamp GR Goble FC. Development of melatonin production in infants and the impact of prematurity. J Clin Endocr Metab. (1992) 75:367–9. 10.1210/jcem.75.2.1639937 1639937

[68] Vilches N Spichiger C Mendez N Abarzua-Catalan L Galdames HA Hazlerigg DG. Gestational chronodisruption impairs hippocampal expression of NMDA receptor subunits Grin1b/Grin3a and spatial memory in the adult offspring. PLoS ONE. (2014) 9:e91313. 10.1371/journal.pone.0091313 24663672 PMC3963867

[69] Schenker S Yang Y Perez A Acuff RV Papas AM Henderson G. Antioxidant transport by the human placenta. Clin Nutr. (1998) 17:159–67. 10.1016/S0261-5614(98)80052-6 10205334

[70] Williams LM Martinoli MG Titchener LT Pelletier G. The ontogeny of central melatonin binding sites in the rat. Endocrinology. (1991) 128:2083–90. 10.1210/endo-128-4-2083 1848509

[71] Soliman A Lacasse AA Lanoix D Sagrillo-Fagundes L Boulard V Vaillancourt C. Placental melatonin system is present throughout pregnancy and regulates villous trophoblast differentiation. J Pineal Res. (2015) 59:38–46. 10.1111/jpi.12236 25833399

[72] Kivelä A. Serum melatonin during human pregnancy. Acta Endocrinol (Copenh). (1991) 124:233–7. 2011913

[73] Redman CW Sargent IL. Latest advances in understanding preeclampsia. Science. (2005) 308:1592–4. 10.1126/science.1111726 15947178

[74] Nakamura Y Tamura H Kashida S Takayama H Yamagata Y Karube A. Changes of serum melatonin level and its relationship to feto-placental unit during pregnancy. J Pineal Res. (2001) 30:29–33. 10.1034/j.1600-079X.2001.300104.x 11168904

[75] Lanoix D Guerin P Vaillancourt C. Placental melatonin production and melatonin receptor expression are altered in preeclampsia: new insights into the role of this hormone in pregnancy. J Pineal Res. (2012) 53:417–25. 10.1111/j.1600-079X.2012.01012.x 22686298

[76] Zeng K Gao Y Wan J Tong M Lee AC Zhao M. The reduction in circulating levels of melatonin may be associated with the development of preeclampsia. J Human Hypertension. (2016) 30:666–71. 10.1038/jhh.2016.37 27251079

[77] Dou Y Lin B Cheng H Wang C Zhao M Zhang J. The reduction of melatonin levels is associated with the development of preeclampsia: a meta-analysis. Hypertens Pregnancy. (2019) 38:65–72. 10.1080/10641955.2019.1581215 30794002

[78] Hannan NJ Binder NK Beard S Nguyen TV Kaitu'u-Lino TJ Tong S Melatonin enhances antioxidant molecules in the placenta, reduces secretion of soluble fms-like tyrosine kinase 1 (sFLT) from primary trophoblast but does not rescue endothelial dysfunction: an evaluation of its potential to treat preeclampsia. PLoS ONE. (2018) 13:e0187082 10.1371/journal.pone.0187082 29641523 PMC5894956

[79] Hobson SR Gurusinghe S Lim R Alers NO Miller SL Kingdom JC. Melatonin improves endothelial function in vitro and prolongs pregnancy in women with early-onset preeclampsia. J Pineal Res. (2018) 65:e12508. 10.1111/jpi.12508 29766570

[80] Arendt J. Melatonin in humans: it's about time. J Neuroendocrinol. (2005) 17:537–8. 10.1111/j.1365-2826.2005.01333.x 16011490

[81] Takayama H Nakamura Y Tamura H Yamagata Y Harada A Nakata M Pineal gland (melatonin) affects the parturition time, but not luteal function and fetal growth, in pregnant rats. Endocr J. (2003) 50:37–43. 10.1507/endocrj.50.37 12733707

[82] Hertz-Eshel M Rahamimoff R. Effect of melatonin on uterine contractility. Life Sci. (1965) 4:1367–72. 10.1016/0024-3205(65)90014-7 5892264

[83] Burns JK. Effects of melatonin on some blood constituents and on uterine contractility in the rat. J Physiol. (1972) 226:106P−7P. 5085309

[84] Abd-Allah AR El-Sayed el SM Abdel-Wahab MH Hamasa FM. Effect of melatonin on estrogen and progesterone receptors in relation to uterine contraction in rats. Pharmacol Res. (2003) 47:349–54. 10.1016/S1043-6618(03)00014-8 12644393

[85] Gimeno MF Landa A Sterin-Speziale N Cardinali DP Gimeno AL. Melatonin blocks *in vitro* generation of prostaglandin by the uterus and hypothalamus. Eur J Pharmacol. (1980) 62:309–17. 10.1016/0014-2999(80)90098-9 6102921

[86] Deng WG Tang ST Tseng HP Wu KK. Melatonin suppresses macrophage cyclooxygenase-2 and inducible nitric oxide synthase expression by inhibiting p52 acetylation and binding. Blood. (2006) 108:518–24. 10.1182/blood-2005-09-3691 16609073 PMC1895491

[87] Glattre E Bjerkedal T. The 24-hour rhythmicity of birth. A populational study. Acta Obstet Gynecol Scand. (1983) 62:31–6. 10.3109/00016348309155754 6858620

[88] Cooperstock M England JE Wolfe RA. Circadian incidence of premature rupture of the membranes in term and preterm births. Obstet Gynecol. (1987) 69:936–41. 3574825

[89] Olcese J Lozier S Paradise C. Melatonin and the timing of human parturition. Reprod Sci. (2012) 20:168–74. 10.1177/1933719112442244 22556015

[90] Beesley S Lee J Olcese J. Circadian clock regulation of melatonin MTNR1b receptor expression in human myometrial smooth muscle cells. Molec Hum Reprod. (2015) 21:662–71. 10.1093/molehr/gav023 25939854

[91] Bunger MK Wilsbacher LD Moran SM Clendenin C Radcliffe LA Hogenesch JB. Mop3 is an essential component of the master circadian pacemaker in mammals. Cell. (2000) 103:1009–17. 10.1016/S0092-8674(00)00205-1 11163178 PMC3779439

[92] Kiyohara YB Tagao S Tamanini F Morita A Sigisawa Y Yasuda M. The BMAL1 C terminus regulates the circadian transcription feedback loop. Proc Natl Acad Sci USA. (2006) 103:10074–9. 10.1073/pnas.0601416103 16777965 PMC1502508

[93] Phaneuf S Rodriguez Linares B Tamby Raja RL MacKenzie IZ Lopez Bernal A. Loss of myometrial oxytocin receptors during oxytocin-induced and oxytocin-augmented labour. J Reprod Fertil. (2000) 120:91–7. 10.1530/jrf.0.1200091 11006150

[94] Olcese J Beesley S. The clinical significance of melatonin receptors in the human myometrium. Fertil Steril. (2014) 102:329–35. 10.1016/j.fertnstert.2014.06.020 25015556

[95] Rahman SA Bibbo C Olcese J Czeisler CA Robinson JN Klerman EB. Relationship between endogenous melatonin concentrations and uterine contractions in late third trimester of human pregnancy. J Pineal Res. (2019) 66:e12566. 10.1111/jpi.12566 30739346 PMC6453747

[96] Brown AG Leite RS Strauss JF. Mechanisms underlying functional progesterone withdrawal at parturition. Ann NY Acad Sci. (2004) 1034:36–49. 10.1196/annals.1335.004 15731298

[97] Menon R Bonney EA Condon J Mesiano S Taylor RN. Novel concepts on pregnancy clocks and alarms: redundancy and synergy in human parturition. Hum Reprod Update. (2016) 22:535–60. 10.1093/humupd/dmw022 27363410 PMC5001499

[98] Olcese J. Circadian aspects of mammalian parturition: a review. Mol Cell Endocrinol. (2012) 349:62–7. 10.1016/j.mce.2011.06.041 21777654

[99] McCarthy R Jungheim ES Fay JC Nates K Herzog ED England SK. Riding the rhythm of melatonin through pregnancy to deliver on time. Front Endocrinol. (2019) 10:616. 10.3389/fendo.2019.00616 31572299 PMC6753220

[100] Waldhauser F Weiszenbacher G Tatzer E Gisinger B Waldhauser M Schemper M. Alterations in nocturnal serum melatonin levels in humans with growth and aging. J Clin Endocrinol Metab. (1988) 66:648–52. 10.1210/jcem-66-3-648 3350912

[101] Reiter RJ. Melatonin and human reproduction. Ann Med. (1998) 30:103–8. 10.3109/07853899808999391 9556096

[102] Sack RL Lewy AJ Erb D Vollmer WM Singer CM. Human melatonin production decreases with age. J Pineal Res. (1986) 3:379–88. 10.1111/j.1600-079X.1986.tb00760.x 3783419

[103] Zhao ZY Xie Y Fu YR Bogdan A Touitou Y. Aging and the circadian rhythm of melatonin: a cross sectional study of Chinese subjects 30-110 yr of age. Chronobiol Int. (2002) 19:1171–82. 10.1081/CBI-120015958 12511033

[104] Magri F Sarra S Cinchetti W Guazzoni V Fiorvantu M Cravello L. Qualitative and quantitative changes of melatonin levels in physiological and pathological aging and in centenarians. J Pineal Res. (2004) 36:256–61. 10.1111/j.1600-079X.2004.00125.x 15066050

[105] Walters JF Hampton SM Ferns GAA Skene DJ. Effect of menopause on melatonin and alertness rhythms investigated in constant routine conditions. Chronobiol Int. (2005) 22:859–72. 10.1080/07420520500263193 16298772

[106] Bellipanni G Bianchi P Pierpaoli W Bulian D Ilyia E. Effects of melatonin in perimenopausal and menopausal women: a randomized and placebo controlled study. Exp Gerontol. (2001) 36:297–310. 10.1016/S0531-5565(00)00217-5 11226744

[107] Amstrup AK Sikjaer T Mosekilde L Rejnmark L. The effect of melatonin treatment on postural stability, muscle strength, and quality of life and sleep in postmenopausal women: a randomized controlled trial. Nutr J. (2015) 14:102. 10.1186/s12937-015-0093-1 26424587 PMC4590707

[108] Toffol E Kalleinen N Haukka J Vakkuri O Partonen T Polo-Kantola P. Melatonin in perimenopausal and postmenopausal women: associations with mood, sleep, climacteric symptoms, and quality of life. Menopause. (2014) 21:493–500. 10.1097/GME.0b013e3182a6c8f3 24065140

[109] Chojnacki C Kaczka A Gasiorowska A Fichna J Chojnacki J Brzozowski T. The effect of long-term melatonin supplementation on psychosomatic disorders in postmenopausal women. J Physiol Pharmacol. (2018) 69:297–304. 30045006 10.26402/jpp.2018.2.15

[110] Yarci Gursoy A Kiseli M Caglar GS. Melatonin in aging women. Climacteric. (2015) 18:790–6. 10.3109/13697137.2015.1052393 26029988

[111] Reiter RJ Tan DX Korkmaz A Rosales-Corral SA. Melatonin and stable circadian rhythms optimize maternal, placental and fetal physiology. Hum Reprod Update. (2014) 20:293–307. 10.1093/humupd/dmt054 24132226

[112] Yang Y Cheung HH Zhang C Wu J Chan WY. Melatonin as potential targets for delaying ovarian aging. Curr Drug Targets. (2018) 20:16–28. 10.2174/1389450119666180828144843 30156157

[113] Tamura H Kawamoto M Sato S Tamura I Maekawa R Taketani T. Long-term melatonin treatment delays ovarian aging. J Pineal Res. (2017) 62:e12381 10.1111/jpi.12381 27889913

[114] Song C Peng W Yin S Zhao J Fu B Zhang J. Melatonin improves age-induced fertility decline and attenuates ovarian mitochondrial oxidative stress in mice. Sci Rep. (2016) 6:35165 10.1038/srep35165 27731402 PMC5059725

[115] Luboshitzky R Qupti G Ishay A Shen-Orr Z Futerman B Linn S. Increased 6-sulfatoxymelatonin excretion in women with polycystic ovary syndrome. Fertil Steril. (2001) 76:506–10. 10.1016/s0015-0282(01)01930-6 11532473

[116] Tagliaferri V Romualdi D Scarinci E Cicco S Florio CD Immediata V. Melatonin treatment may be able to restore menstrual cyclicity in women with PCOS: a pilot study. Reprod Sci. (2018) 25:269–75. 10.1177/1933719117711262 28558523

[117] Jamilian M Foroozanfard F Kavossian MN Aghadavod Stadmohammadi V Kia M Eftekhar T. Effects of melatonin supplementation on hormonal, inflammatory, genetic, and oxidative stress parameters in women with polycystic ovary syndrome. Front Endocrinol. (2019) 10:273 10.3389/fendo.2019.00273 31139144 PMC6527800

[118] Song X Sun X Ma G Sun Y Shi Y Du Y. Family association study between melatonin receptor gene polymorphisms and polycystic ovary syndrome in Han Chinese. Eur J Obstet Gynecol Reprod Biol. (2015) 195:108–12. 10.1016/j.ejogrb.2015.09.043 26519818

